# Stem Cell Homing in Intrathecal Applications and Inspirations for Improvement Paths

**DOI:** 10.3390/ijms23084290

**Published:** 2022-04-13

**Authors:** Dusan M. Maric, Gordana Velikic, Dusica L. Maric, Gordana Supic, Danilo Vojvodic, Vedrana Petric, Dzihan Abazovic

**Affiliations:** 1 Department for Research and Development, Clinic Orto MD-Parks Dr Dragi Hospital, 21000 Novi Sad, Serbia; ducamaric@gmail.com; 2 Faculty of Dentistry Pancevo, University Business Academy, 26000 Pancevo, Serbia; 3 Vincula Biotech Group, 11000 Belgrade, Serbia; adzihan@gmail.com; 4 Department of Anatomy, Faculty of Medicine, University of Novi Sad, 21000 Novi Sad, Serbia; 5 Institute for Medical Research, Military Medical Academy, 11000 Belgrade, Serbia; gogasupic@gmail.com (G.S.); vojvodic.danilo@gmail.com (D.V.); 6 Medical Faculty of Military Medical Academy, University of Defense, 11000 Belgrade, Serbia; 7 Infectious Diseases Clinic, Clinical Center of Vojvodina, 21000 Novi Sad, Serbia; vedrana.petric82@gmail.com; 8 Department of Infectious Diseases, Faculty of Medicine, University of Novi Sad, 21000 Novi Sad, Serbia; 9 Department for Regenerative Medicine, Biocell Hospital, 11000 Belgrade, Serbia

**Keywords:** stem cells, intrathecal administration, neurodegenerative diseases

## Abstract

A transplanted stem cell homing is a directed migration from the application site to the targeted tissue. Intrathecal application of stem cells is their direct delivery to cerebrospinal fluid, which defines the homing path from the point of injection to the brain. In the case of neurodegenerative diseases, this application method has the advantage of no blood–brain barrier restriction. However, the homing efficiency still needs improvement and homing mechanisms elucidation. Analysis of current research results on homing mechanisms in the light of intrathecal administration revealed a discrepancy between in vivo and in vitro results and a gap between preclinical and clinical research. Combining the existing research with novel insights from cutting-edge biochips, nano, and other technologies and computational models may bridge this gap faster.

## 1. Introduction

Stem cells are essential to the regenerative processes with a primary function to replace damaged cells in the body. Their ability to differentiate into functional cells is an outcome of their response to micro-environmental changes, i.e., stem cells’ interaction with signals released by the affected tissue. Stem cells reside in many tissues and organs, including fat, bone marrow, liver, brain, or umbilical cord—blood and lining. Medical use of stem cells requires their isolation with an end goal to culture sufficient quantities of stem cells for therapeutic effect. A stem cell application refers to the transplantation of stem cells with the ultimate intention to reach the targeted area. There are two fundamental issues to consider before each stem cell application. The first issue refers to methods that will enable the stem cell transformation to targeted cells or successful engrafting. The second issue is how to direct the migration of most of the transferred cells to the desired location. There are many proposed solutions to the first issue, and they are not the subject of this digested review. The second issue is the subject of cell homing. It is logical to conclude that the higher the number of administered cells or administration closer to the targeted site, the higher is the homing likelihood success of transplanted cells. Currently, we know that the greater the number of administered stem cells, the better the treatment outcomes. However, the number of delivered cells has a saturation plateau, after which no additional treatment enhancement is noted [1]. Understanding the intrinsic mechanisms of cell homing may be essential to increase the success of the stem cell-based treatment and accomplish more with a reduced number of the administered cells.

For a spectrum of neurodegenerative diseases, such as, but not limited to, Alzheimer’s disease, autism, stroke, Parkinson’s disease and Huntington’s disease, or multiple sclerosis, the targeted area is in the brain. The literature is abundant with evidence of positive results for non-invasive methods of stem cell transplant [2,3,4]; however, parenteral routes of administration typically result in dissipation of stem cells to other organs rather than the brain. Intrathecal space is also known as subarachnoid space. It is a space between the membranous layers of the arachnoid matter and pia matter and surrounds the brain and the spinal cord, filled with cerebrospinal fluid (CSF). Intrathecal administration is a preferred method of drug delivery when the blood–brain barrier (BBB) restricts the delivery to the brain [5,6], for example, via oral or parenteral administration. In terms of homing for neurodegenerative diseases, this may be a preferred choice of application because of lesser dissipation of stem cells due to the anatomical predisposition of the intrathecal administration point.

## 2. Cell Homing

### 2.1. A Definition

Cell homing is a stem cell’s ability to find a point of destination, be it a tissue in distress or a niche. Cell homing is a diverse term. Many cell types, including stem cells, progenitors, and mature, specialized T cells, are homing to their respective niches—environments that promote their self-renewable state. Note that our focus is on stem cell transplantation with the ultimate goal of the remission of neurodegenerative diseases. The purpose frames the context for a definition. Thus, we may adopt that cellular homing implicates a mechanism by which a location of damage releases signaling molecules that start recruitment, proliferation, migration, and the differentiation of stem and progenitor cells. It is an endogenous mechanism that drives stem cells from their niches to a site of injury or inflammation to respond to signals coming from damaged areas. Transplanted stem cell homing is a directed migration from the application site to the targeted location. This definition illustrates that for stem cell-based therapies, it is imperative to understand the mechanisms that increase the possibility of the controlled directing of the stem cells to the targeted tissues [7]. The question at hand—“How to improve cell homing?, comes down to methods that can improve the “attractiveness” of the targeted location to administrated cells and prevent their dissipation to unintended sites. For better clarity of research results of homing mechanisms, it is crucial to establish a common ground between results of different research groups or standardize protocol aspects. Yusuf et al. outlined a classic protocol on homing of hematopoietic stem cells (HSC) to the bone marrow (BM), with procedures that adapt to a design and a goal of an experiment [8]. Protocols tailor different points of interest, such as cytokine release or study of the homing outcome with no induced injuries.

### 2.2. Homing Evidence Following Intrathecal Application

The result’s assessment of in vivo clinical trials of stem cell intrathecal applications targets the side effects, feasibility, safety, and visible and measurable health improvements after the application [9,10,11]. The assumption is that in the case of intrathecal application in humans, (most of) the injected cells will migrate to the central nervous system (CNS) lesion or affected area in the brain. Compared to other application methods, guided neurosurgical delivery is superior in the function of the absolute cell count reaching the brain. However, it comes at the risk of focal bleeding, with the intrathecal application as a good, if not a primary, alternative [12].

Several clinical studies demonstrated that serious adverse events of intrathecal applications are rare; instead, they have mild and temporary side effects. The health improvements are present in different degrees, many with confirmed values of monitored parameters within limits of other studies and with an overall focus on the procedure’s success, feasibility, and safety [13], even on five-year follow-up [14]. Reported complications are headache [15], potential risk of hydrocephalus, and lumbosacral radiculopathies [16,17]. Phase I/II study on the tolerability of MSCs intrathecal transplantation in patients with early multiple system atrophy showed that intrathecal is as safe as intravascular administration, with mild side effects at higher doses. The efficacy was slightly lower compared to historical groups. They also registered neurotrophic factors in CSF, indirectly demonstrating the occurrence of homing [18].

An initial number of molecules of substances delivered via intrathecal administration may decrease due to CSF’s role in facilitating waste products [19]. The intrathecal administration shortens the path to the target location in the brain and thus lowers the unwanted dissipation of transferred stem cells. Hence, intrathecal administration may be a preferred choice in terms of efficiency for cell migration to the desired location. However, Kim et al. reported that only 2.4% of the intrathecally injected Wharton’s Jelly-derived MSCs (WJ-MSCs) reached the rat’s brain. Their study was the first to measure the ratio of homing cells at the target location. A tenfold dosage increase resulted in increased homing efficiency by 2.6. They also concluded that the migration time is between 6 and 12 h, indicating that the human’s homing time is probably longer because of the anatomical differences. They argue that most stem cells never left the lumbar area since they did not migrate to other organs and that the increased homing rate at higher dosage is due to a higher clumping tendency on higher concentrations, which made them robust to CSF clearing [20].

Although Kim et al. reported no migration to other organs 12 h after injection, Quesada et al. detected dissipation to mice heart 24 h later, and brain and heart four months later, thus providing evidence that homing exceeds the intended location given sufficient time [21].

Barberini et al. tracked the technetium radiolabeled MSCs and reported better distribution of MSCs within the subarachnoid space [22]. They also observed no trace of MSCs at the damaged tissue within a week after injection. Vaquero et al. reported arrest and improvement of Alzheimer’s type dementia symptoms and indirect homing evidence in the form of increased cerebral glucose metabolism. They measured cerebral glucose metabolism via 18F-fluorodeoxyglucose positron emission tomography after the administration [11].

One study demonstrated that the ratio of MSCs at the injured site vs. intact spinal cord was significantly high to conclude that MSCs primarily migrate to the injured area [23]. The same research paper also noted that some cells migrated to perivascular spaces of damaged tissue. Oh et al. administrated repeated intrathecal injections one month apart in two trials. They noted no acceleration in amyotrophic lateral sclerosis functional rating scale-revised in the first study and change from baseline to four at six months follow-up in the second study [24]. A. Sahrain et al. injected a booster dose after a year, with no adverse effects reported and a notable improvement in health progression [25]. Kuang et al. administered four intrathecal injections, a week apart each, with no adverse effects and with observable improvements in health [13]. None of these studies compared the improvements to the case of a single dose, partially because it was not feasible due to the studies’ designs. However, they all indirectly confirmed the homing effect in relative health improvement and verified the treatments’ feasibility and safety. 

## 3. Discussion and Future Perspectives 

### 3.1. Knowledge Quest for Homing Mechanisms: Enhancing the Homing Efficiency

#### 3.1.1. Questioning the Homing Impact on Cell Therapy Results: The Need for Metrics

Until the 1960s, the scientific dogma was that the adult brain neither produces new neurons nor regenerates. This paradigm changed with the evidence of the neural stem cells (NCSs) in the adult brain, generated throughout the brain’s lifetime. In vitro, NCSs are activated and differentiated into neurons, astrocytes, and oligodendrocytes upon brain trauma. The identified NCSs niches are in the subventricular zone of the forebrain and the subgranular zone of the dentate gyrus within the hippocampus [26]. However, NCSs are not a prime choice for administration because they are hard to harvest and tend to transform into cancer cells. While all stem cell types are under scientific investigation, the most popular are pluripotent bone marrow (BM)-derived mesenchymal stem cells (MSCs). The MSCs’ popularity is due to their ability to secrete neurotrophic factors and differentiate into neuron-like cells. They also have well-researched immunomodulatory properties, homing capacity, multilineage differentiation, and beneficial experimental outcomes in various animal models of neurological diseases. Most studies currently focus on the use of intravenous administration and rely on cells’ capacity for targeted migration, i.e., “homing”, and the secretion of cytokines, growth factors, and immunomodulatory correction mechanisms. Research shows CSF transports the stem cells administrated into the subarachnoid space of the lumbar segment of the spinal cord and further in the subarachnoid space of the brain. Thus, in cases of neurodegenerative diseases, these cells have a higher impact on affected areas, unlike intravenous applications, which have limited access to the CNS due to the BBB [6]. The only exception is the use of umbilical cord blood autotransfusion in newborns with clinical and laboratory signs of hypoxia. The BBB is open in newborns and passes embryonic cells with high-potency stem cells in the brain structure.

Stem cell-based therapy is beneficial in treating chronic conditions with neurological deficits by nerve cell replacement, activating polymodal synapses, increasing the efficiency of the existing synapses, or stimulating the development of new blood vessels. Scientific literature search shows that experiments and studies confirm the homing effect for stem cells transplanted via the intrathecal application. However, a method is needed to measure the quantitative efficiency of stem cells, i.e., how many cells eventually reached and were effective at the lesion, in the function of the subject’s demography and lesion type and location, or neurodegenerative disease. The missing information is the ratio of administrated cells that dissipated into other organs and the dissipation locations in real-time. Designing such metrics is not an easy task. Due to various technology and mandatory limits, i.e., technical, ethical, and methods maturity—the answers to most of our ambitions for a higher clarity on the homing mechanisms come from the results of preclinical studies. Although in vitro research has provided valuable insights on a homing molecular basis, they cannot fully clarify mechanisms at work in vivo. In vivo stem cell homing imaging is essential for understanding the cells’ activities, interactions, and homing mechanisms [27].

#### 3.1.2. Who Sends the Invitations?

According to [20], a high percentage of stem cells do not migrate to the desired location. Higher doses or multiple applications may boost this number [28,29]. Nevertheless, this result also encourages further investigations of the homing mechanism and its enhancement. There is considerable evidence that different methods may enhance homing efficiency. The frequently investigated methods include: (1) chemical factors that regulate cells’ migration, (2) mechanical factors, and (3) manipulations of the targeted tissue. It is interesting to observe that methods provide different approaches; however, trigger similar, if not the same, consequences.

##### Homing at Molecular Levels: Invitations via Chemical Factors

Several research groups found that the interference with chemokines that stimulate the cells’ migration raises homing efficiency. One of the first to be identified is stromal-derived factor 1 (SDF-1)—a chemokine, and its receptor, CXCR4, the so-called SDF-1/CXR4 axis. High levels of SDF-1 support MSCs’ homing. In [30], researchers created a chemokine called S-SDF-1(S4V)—a version of SDF-1, but robust to protease deactivation, including metalloproteinase-2. Direct injection of this version of SDF-1 into the injured heart of a rat attracted more stem cells to the damaged heart and improved the heart’s functions. Other research groups used different drugs or hormones to maintain the activity of SDF-1, which increased the recruitment of stem cells to a damaged heart and resulted in the heart’s improved function [31,32,33,34,35,36,37]. Soon it has become apparent that research needs to extend the focus to the different axis of chemokines and to include other receptors, for example, CXCR7, or other axes, such as CCL27-CCR10 and CCL21-CCR7 [38,39]. These methods give insights into homing mechanisms and enable the potential to choose the best strategies to amplify chemokine signals that affect homing, as homing success is connected to relevant cell surface homing ligands. It also directed the research of homing signaling molecules to optimize in situ regeneration. This approach can improve the homing process of both endogenous and exogenous processes and enhance the cell number and therapeutic procedure at the site of damage. In addition, therapies based on homing signaling molecules may bypass the need for external administration or decrease the needed number of cells, thus reducing the costs of both—time and labor—of the isolation of stem cells.

Genetic modifications and surface glycoengineering may also keep stem cell surfaces more susceptible to homing factors. R. Sackstein and his team proved that converting native CD44, a key homing molecule for MSC, to sialofucosylated glycoform of CD44, improves MSCs trafficking through interactions with E-selectin and, thus, navigates MSCs to their BM niche more effectively [40]. Karp and his team accomplished similar results with in vitro ‘priming’ of MSCs in the presence of various factors that increased the expression of cell surface chemotactic receptors and migratory capacity of MSCs [41]. Adams et al. reported that guanine-nucleotide-binding stimulatory α subunit (GαS) protein transmembrane signaling is needed to enable HSCs homing to their niches. The HCSs homing increased after GαS pharmacological treatment with cholera toxin, and the toxin irreversibly activated GαS via inhibition of GαS’ intrinsic GTPase activity [42].

Growth factors are proteins that promote growth, cell migration, differentiation, organization, and maintenance of cells and tissues. Their ability to promote migration puts them on the radar of homing research. Many growth factors have been reported to enhance MSCs migration. However, several of the most pivotal are vascular endothelial growth factors (VEGFs) with their receptor VEGFR—which stimulate platelet-derived growth factor receptors and MSC migration; and platelet-derived growth factor (PDGF) and insulin-like growth factor 1 (IGF-1)—both of which stimulate chemotaxis of MSC cells [43,44]. 

In addition, it has been reported that chemical compounds found in medicinal plants impact signaling pathways that enhance homing, including already mentioned different axis of chemokines and growth factors [45].

##### Homing at Physical Levels: Invitation via Mechanical Factors

Mechanical factors are a direct consequence of the microenvironment where migration occurs, including stiffness, strain, and shear stress. Mechanical stretching of 10% during 12 h activated SDF-1α/CXCR4 axis and increased MSC homing, while a hemodynamic force of shear stress of 0.2 Pa activated Jun N-terminal kinase (JNK) and p38 mitogen-activated protein kinase (MAPK) pathways and raised the homing rate of MSCs [46,47]. It is known that CSF has natural pulsations, and the injected volume may influence spinal canal intrinsic pressure, thus directly affecting the mechanical properties of its environment. However, the injection methods either minimize the changes or may induce undesirable effects due to the increased intracranial pressure [48].

##### Raising the “Attractiveness” of Targeted Sites: The Postcard from Home

Researchers also focused on the properties of targeted tissues. They increased the homing attractiveness of the tissue by changing its properties with the aid of ultrasound, magnetic and electric fields, and irradiation. Better homing after irradiation is attributed to a noted increase along the axis SDF-1/CXCR4 [49]. Both natural and external electric fields (EFs) influence the migration, and longer EFs in vitro exposure demonstrated migration [50]. Magnetic targeting has a higher impact if the cells are labeled with magnetic nanoparticles, such as iron oxide. The labeling increased secreting across the CCR1, and CXCR4 axes, thus raising the migration of MSCs. Even in the absence of the external magnetic field, the effect existed with an expression of migration-related proteins c-Met and C-C motif chemokine receptor 1 [51,52,53]. Sonic manipulation proved to increase MSC homing multifold. Pulsed focused ultrasound promotes local gradient of cytokines (SDF-1α, IL-1α, IL-1β, and MCP-1), growth factors (VEGF, FGF, HGF, and PLGF), and adhesion molecules (ICAM-1, VCAM-1), all of which enhance MSCs homing [54,55].

### 3.2. Back to the Future

#### 3.2.1. A Little Help from the Friends: Paracrine Signaling and Microvesicles

The therapeutic action of stem cells is attributed to the secretion of bioactive and growth factors, which promote neurogenesis, angiogenesis, and vasculogenesis. In addition, scientific literature grows in evidence that exosomes released from MSCs via paracrine signaling have positive effects on neurodegenerative diseases with the same effects as MSCs, if not even alone responsible for MSCs therapeutic effect, and without cell-therapy side effects [56,57,58,59,60,61]. Exosomes are nano-sized extracellular vesicles that take part in cell–cell communication. They carry a cargo that includes a unique content of proteins, various ribonucleic acids (RNAs)—including micro-RNA, and lipids. Note that micro RNAs are included in controls of neural remodeling, angiogenesis, and neurogenesis processes. Although many studies have analyzed the health benefits of the exosomes sole deployment, exploring the exosome role in the MSCs homing would be beneficial [62,63,64,65].

#### 3.2.2. Vitality: Let the Life Force Be with Stem Cells

Lee et al. suggested cultivating MSCs in the CSF of a patient, prior to the implantation, as a method to increase the efficacy of MSCs, preconditioning them to the CSF microenvironment and, thus, increasing MSCs’ robustness to the CSF flow clearance mechanism [66]. The viability of stem cells is vital for the efficacy of the cell treatment because it determines the percentage of live cells in a stem cell culture. Low viability values may influence many stem cell therapy mechanisms, including homing. The math seemed simple—more cells—the higher probability of homing efficacy. Conversely, lowering the concentration increased the widespread migratory capabilities and the viability of intracerebroventricular-delivered human mesenchymal stem cells. On the other hand, low concentrations of MSCs may result in an insufficient number of cells remaining after washout that could generate therapeutic effects, but this could be compensated by performing multiple treatments. Any manipulation, such as increasing the stem cell homing ability or enabling stem cell monitoring, must not influence the colony’s viability [67,68,69,70].

#### 3.2.3. In Vivo Labs

Levin et al.’s research demonstrated the importance of signals temporal patterns for regeneration [71]. They chemically controlled intrinsic electric fields of planaria via the manipulation of ion channels and electrical synapses and managed to control the regeneration outcome. They also observed that micromanipulation is not crucial because the cells automatically perform micromanagement of the processes once the process starts. Thus, another pathway to increase stem cells’ homing receptivity may be a time-changing signal that “nudges” the cells to targeted locations and prevents their dissipation to other areas. It is crucial to notice that visualization of the processes needs to be backed with understanding cells’ interactions at the molecular level. In addition, the tissue nanotransfection technology (TNT) enables in vivo, half-an-hour-long reprogramming of one tissue type to another with a harmless electric discharge [72]. TNT is tested on mice with induced brain stroke to non-virally deliver fibroblasts with developmental transcription factor genes (Etv2, Foxc2, and Fli1). They succeeded in reprogramming in vivo stromal tissue into new vascular tissue. TNT is a non-invasive method and deployable at the point of care. The technology can potentially navigate and increase the mobility of stem cells or raise the homing attractiveness of the targeted area; although, the technology is currently not deployed on stem cells [73,74,75].

#### 3.2.4. Interdisciplinary Going Multidisciplinary

In the years of the rapid growth of computational power, available data, or success of artificial intelligence models, and their penetration in multidisciplinary research, it would be unreasonable to think that stem cell computational modeling would be an exception. However, the stem cell computational models are still not receiving the proper attention in the stem cell research community. The models may be essential in understanding the missing links in the homing mechanisms [76]. The use of models would lead to broader benefits, such as overcoming the limits of in vivo protocols, a better understanding of the impact of in vitro results on in vivo trials, better design of the stem cell transplantation therapies, better insight into research results, and more appropriate prediction of the outcome of the experiments [77]. In addition, it will open a possibility to a shorter time from experiments’ results to its deployment. Computer simulations of stem cell intracellular signal communication may provide better insight into key-signaling molecules in homing mechanisms and identify clues for better transplantation efficiency. The benefits are bidirectional because computing techniques and tools often deploy knowledge acquired from the biomedical field. For example, the biological base of the functioning of neural cells was the inspiration for neural network computing methods. Note that the homing model for intrathecal administration is “simpler”, as the “first draft” does not require modeling the processes before the BBB and the BBB crossing [78,79,80]. In addition, a combination of good in vitro 3D models, such as the potential of bio-electrospray [81] for spatial modeling and comprehensive molecular computational models, may be a winning combination to clarify the homing mechanisms.

## 4. Conclusions

Many good reviews and research papers have been written on stem cell homing within the last two decades. The take-home lessons they highlight are 1. The stem cell injection site impacts the homing results. The closer the injection to the targeted location—the better migration results; 2. Preprocessing may increase homing efficiency; 3. There is a number of potential methods that may improve the homing mechanisms; 4. The mechanism of a neurodegenerative disease is essential to understand the homing processes and predict the engraftment results; 5. Stem cells mainly improve the plasticity of the brain; although, the process needs further investigation; 6. MSCs are the most common choice; 7. An intrathecal application has many benefits, fewer adverse effects, and is generally accepted as safe. It is a good choice of administration for neurodegenerative diseases due to low invasiveness and no BBB; 8. Novel findings imply that the paracrine signaling of stem cells may shift research focus to stem cells’ secreted paracrine factors and microvesicles. As a result, exosomes are gaining in popularity, particularly for parenteral routes of administration; 9. The discrepancy between in vivo and in vitro results needs further investigations; 10. The researchers need to pay more attention to the prospects of mathematical, physical, and computer models and simulations; 11. Real-time development and spatial information of homing processes need profound investigation regardless of the method of administration. 12. Homing mechanisms in intrathecal and other ways of administration remain not adequately understood, with a need for further elucidation. 

The observation of injected cells migration effects in mice, which emerged after four months, emphasized the importance of long-term studies [20]. However, this opens new questions. For example, how much is the long-term effect affected by the complexity of the autonomic nervous system, which regulates circadian endogenous homing response [82,83,84,85], or what slows down the migration of injected cells?

## Data Availability

Not applicable.
